# Integrated Physiochemical, Hormonal, and Transcriptomic Analysis Revealed the Underlying Mechanisms for Granulation in Huyou (*Citrus changshanensis*) Fruit

**DOI:** 10.3389/fpls.2022.923443

**Published:** 2022-07-14

**Authors:** Chen Kang, Anze Jiang, Han Yang, Guixia Zheng, Yue Wang, Jinping Cao, Chongde Sun

**Affiliations:** ^1^Laboratory of Fruit Quality Biology, The State Agriculture Ministry Laboratory of Horticultural Plant Growth, Development and Quality Improvement, Horticultural Products Cold Chain Logistics Technology and Equipment National-Local Joint Engineering Laboratory, Zhejiang Provincial Key Laboratory of Integrative Biology of Horticultural Plants, Zhejiang University, Hangzhou, China; ^2^Quzhou Kecheng District Chai Family Citrus Professional Cooperative, Quzhou, China

**Keywords:** cell wall components, citrus, juice sac granulation, phytohormones, transcriptomic analysis

## Abstract

Juice sac granulation is a common internal physiological disorder of citrus fruit. In the present study, we compared the physiochemical characteristics and transcriptome profiles of juice sacs in different granulation levels from Huyou fruit (*Citrus changshanensis)*. The accumulation of cell wall components, including the water-soluble pectin, protopectin, cellulose, and lignin, were significantly correlated with the granulation process, resulting in the firmness increase of the juice sac. The *in situ* labeling of the cell wall components indicated the early accumulation of cellulose and high-methylesterified pectin in the outer layer cells, as well as the late accumulation of lignin in the inner layer cells of the juice sac. Several phytohormones, including auxins, abscisic acids, cytokinins, jasmonic acid, salicylic acid, and/or their metabolites, were positively correlated to the granulation level, indicating an active and complex phytohormones metabolism in the granulation process. Combining the trend analysis by the Mfuzz method and the module-trait correlation analysis by the Weighted Gene Co-expression Network Analysis method, a total of 2940 differentially expressed genes (DEGs) were found to be positively correlated with the granulation level. Gene Ontology (GO) enrichment indicated that the selected DEGs were mainly involved in the cell wall organization and biogenesis, cell wall macromolecule metabolic process, carbohydrate metabolic process, and polysaccharide metabolic process. Among these selected genes, those encoding β-1,4-xylosyltransferase IRX9, cellulose synthase, xyloglucan: xyloglucosyl transferase, xyloglucan galactosyltransferase MUR3, α-1,4-galacturonosyltransferase, expansin, polygalacturonase, pectinesterase, β-glucosidase, β-galactosidase, endo-1,3(4)-β-glucanase, endoglucanase and pectate lyase that required for the biosynthesis or structural modification of cell wall were identified. In addition, NAC, MYB, bHLH, and MADS were the top abundant transcription factors (TFs) families positively correlated with the granulation level, while the LOB was the top abundant TFs family negatively correlated with the granulation level.

## Introduction

Juice sac granulation is a common internal physiological disorder in citrus fruit, which is characterized by the hard and granulated appearance, as well as the juiceless and flavorless taste of juice sacs. Juice sac granulation happens in various citrus types, with the incidence of 9.2–18.6% in *Citrus reticulate*, 7.2–14.3% in *C. sinensis*, 6.7–8.7% in *C. limon*, 12.6–16.2% in *C. paradisi*, and 11.3– 17.9% in *C. maxima*, as is reported in the investigation of Sharma et al. (2006). At present, juice sac granulation is still a difficult problem to be solved in the citrus industry.

The causes for the juice sac granulation seem to be various and complex. The genetic background is an important determinant of the granulation index (El-Zeftawi et al., 1985; Daulta and Arora, 1990; Sharma et al., 2006). The inappropriate irrigation and fertilization strategies also lead to granulation (Shiraishi et al., 1999; Ritenour et al., 2004). The fruit harvested over-mature tend to develop granulation (Burns and Albrigo, 1998). In addition, it is found from the productive experiences that many factors, such as an inappropriate leaf-fruit ratio, fruit position, and tree age, might lead to granulation.

To date, several studies had been performed to explore the underlying mechanisms of juice sac granulation. Yao et al. (2018) reported that the alteration of sugars and organic acid metabolism that occurred in granulated juice sacs might be the main cause of granulation in Ponkan fruit. By comparing the healthy and granulated juice sacs, Yao et al. (2020) found the molecular changes in juice sac granulation at transcriptome and DNA methylation levels. In order to investigate the molecule evens that happened in the granulation process, Shi et al. (2020) used the *in vitro* cultivated juice sacs as materials. They found that the lignification was involved in the granulation of the pomelo juice sac, and an MYB transcription factors (TF) CgMYB58 positively regulated the lignin biosynthesis genes in the granulation, which were induced by abscisic acid (ABA) while repressed by indole acetic acid (IAA). Beyond the referred publications, there are relatively few studies on the molecule mechanisms of juice sac granulation, due to the complexity and invisibility of the syndromes.

The juice sac granulation syndromes varied in different citrus species. Our previous study reported the vesicle dying syndromes of Shatangju fruit, with the juice sac collapsing in the late stage (Cao et al., 2020). We also found that the large-size fruit tended to develop granulation (Kang et al., 2022). Huyou (*Citrus changshanensis*) is one of the Chinese native citrus cultivars, in which the juice sac granulation is commonly seen in the ripe fruit. In the pilot study, we found that, although the Huyou fruit had a granulate appearance in the disordered juice sac like other pomelo fruit, no dramatic increase of lignin content can be detected in the early stage of granulation. These indicated that there might be different mechanisms of granulation in different citrus species. In the present study, we compared the physicochemical characteristics and the transcriptome profiles of Huyou juice sacs in different granulation levels and identified candidate transcripts related to the juice sac granulation (especially the early events) through the combination of expression trend analysis and co-expression network construction.

## Materials and Methods

### Plant Materials

Huyou (*C. changshanensis*) fruit was harvested on December 8, 2020, from a local orchard in Changshan County, Zhejiang province, China. The fruit with transverse diameters lower than 90 mm and higher than 105 mm were collected as small-size fruit and large-size fruit, respectively. The healthy juice sacs collected from small-size fruit were named S. The juice sacs from large-size fruit were divided into four groups according to their granulation level: L1 (normal), L2 (slightly granulated), L3 (mediumly granulated), and L4 (severely granulated). The peel of approximately 1 cm wide at the equatorial plane was taken from small-size and large-size fruit and named SP and LP, respectively. All the samples were frozen in liquid nitrogen immediately and stored at –80°C for later use. Three biological replicates were done for each group, with the mixture of tissues from 15 fruit as one biological replicate.

### Physiochemical Indexes Determination

The rind color was measured using a colorimeter (Hunterlab, Reston, VA, United States), at four evenly distributed equatorial sites of each fruit. The CIE 1976 L * * b * color scale was adopted and the citrus color index (CCI) of each fruit was calculated as a* × 1000/(b* × L*) (Cao et al., 2019). The total soluble solids (TSS) were measured with a refractometer PR101-a (Atago, Tokyo, Japan) with two measurements per fruit. The edible rate was calculated as the weight percentage of the pulp. The shape index was calculated as the ratio of longitudinal diameter to the transverse diameter. The granulation index was evaluated based on the method of Chen et al. (1995) and Nie et al. (2020). The granulation level of the fruit was evaluated according to the portion of the granulated area: No granulation, level 0; granulated area ≤ 10%, level 1; 10% < granulated area ≤ 25%, level 2; 25% < granulated area ≤ 50%, level 3; granulated area > 50%, level 4. The granulation index was calculated according to the following formula: (ΣGranulation level × Number of fruit in this level)/(The highest granulation level (level 4) × Total fruit number). A total of 30 small-size or large-size fruit were used respectively for the above analysis.

The firmness of the juice sac was determined using a texture analyzer (Texture Technologies, Hamilton, MA, United States) equipped with a P2 probe, and the parameters were set as follows: pre-test speed of 3 mm/s, test speed of 2 mm/s, post-test speed of 3 mm/s, test distance of 2 mm and trigger force of 0.02 N. The value of firmness was represented as maximum compression force (N). The whiteness index (WI) of the juice sac was measured using the colorimeter (Hunterlab, Reston, VA, United States) and calculated as 100 – [(100 – L*)^2^ + a^*2^ + b^*2^]^0^.^5^ (Amanatidou et al., 2000). A total of 30 juice sacs replicates were done for each group in the above analysis. The water content and the proportion of dry matter were calculated according to the water loss after lyophilization, as was described in the Supplementary Method 1. Three replicates were done for each group, with a mixture of samples from 15 fruit in each replicate.

### Soluble Sugars, Organic Acids, Amino Acids, and Cell Wall Components Contents Analysis

The lyophilized samples were ground into fine powder for the qualitative and quantitative analysis of soluble sugars, organic acids, amino acids, and cell wall components. Three replicates were performed for each group, with 15 fruit in each replicate. The gas chromatography-mass spectrometer (GC-MS) technique was applied for the identification and qualification of soluble sugars, organic acids and amino acids, according to the method of Kang et al. (2022). The contents of soluble sugars, organic acids, and amino acids were calculated by the standard curves of authentic standards (Solarbio, Beijing, China). The Alcohol insoluble solids (AIS) contents were determined according to the method of Dietz and Rouse (1953). The water-soluble pectin and protopectin were isolated and quantitated according to the method of Dong et al. (2008). The cellulose and hemicellulose were isolated and quantitated according to the method of Zhou et al. (2011) with slight modification. The lignin was isolated and quantitated according to the method of Chang et al. (2008). Three replicates were done for each group, with a mixture of samples from 15 fruit in each replicate. (Refer to Supplementary Method 1 for details regarding the protocol).

### Cell Wall Components Labeling in the Histologic Section of Juice Sac

For the *in situ* labeling of lignin, the juice sacs were cut transversely and longitudinally respectively, and the lignin was stained with phloroglucinol. For the histiocyte labeling of pectin, cellulose, and lignin, the paraffin sections of juice sacs were prepared according to the protocol of Li et al. (2017) with slight modifications. The pectin was labeled by immunofluorescence staining with JIM5 and JIM7 (Plant Probes, Leeds, United Kingdom) for the labeling of low-methylesterified pectin and high-methylesterified pectin, respectively, according to the method of Chudzik et al. (2018) with slight modification. The cellulose was stained with Calcofluor White (Coolaber, Beijing, China) according to the manufacturers’ instructions and observed in the excitation wavelength of 405 nm. The lignin was stained with toluidine blue solution and observed under brightfield. (Refer to Supplementary Method 1 for details regarding the protocol).

### Phytohormones Metabolites Profiling

The relative quantities of phytohormones metabolites were analyzed by Wuhan Metware Biotechnology Co., Ltd (Wuhan, China) based on the liquid chromatography-electrospray ionization-tandem mass spectrometry (LC-ESI-MS/MS) according to the method of Chen et al. (2013) with slight modification. The contents of phytohormones were calculated using the external standard method and expressed as ng/g on a fresh weight basis. Three replicates were done for each group, with a mixture of samples from 15 fruit in each replicate. (Refer to Supplementary Method 1 for details regarding the protocol).

### RNA Extraction, Sequencing, and RNA-seq Data Analysis

The total RNA was extracted using a CTAB-pBIOZOL reagent according to Liu et al. (2006). Approximately 80 mg of sample was used for extraction, and the RNA was qualified by a NanoDrop and Agilent 2100 bioanalyzer (Thermo Fisher Scientific, Waltham, MA, United States). Three biological replicates were conducted for each group, with a mixture of samples from 15 fruit in each replicate.

The library construction and RNA sequencing were performed by BGI (Wuhan, China). The constructed libraries were sequenced by the Illumina HiSeq 4000 sequencing platform to obtain a clean raw read. After filtering with SOAPnuke (Version 1.4.0), all clean reads were mapped to the reference genome [*Citrus sinensis* genome v2.0 (HZAU) GCF_000317415.1_Csi_valencia_1.0^1^].

The expression levels of genes were estimated by the values of fragments per kilobase of transcript per million mapped reads (FPKM). Differential expression analysis was performed using the DESeq2 (Version1.4.5) with *Q* value ≤ 0.05 (Love et al., 2014). The differentially expressed genes (DEGs) between two groups were restricted with a false discovery rate (FDR) < 0.05 and | log2(fold change) | > 1. All the transcripts were annotated based on the following public databases: Nr (NCBI non-redundant protein sequences), GO (Gene Ontology), and KEGG (Kyoto Encyclopedia of Genes and Genomes). The trends of DEGs expression were analyzed by the Mfuzz software package of the R-package. The co-expression network construction was conducted by the Weighted Gene Co-expression Network Analysis (WGCNA) package of the R-package.

### Quantitative Real-Time PCR

The total RNA was extracted using a CTAB-pBIOZOL reagent according to Liu et al. (2006). First-strand cDNA synthesis and Quantitative Real-Time PCR (RT-qPCR) analyses were carried out according to the protocol reported by Liu et al. (2020), with primers listed in Supplementary Table 1. Three independent biological replicates were done for each sample.

### Statistical Analysis

All samples were prepared and analyzed with at least three repetitions. Data were statistically assessed by SPSS (Version 20.0, United States). Statistical significance of differences was calculated using Tukey multiple range test or *t*-test, with *p* < 0.05 considered as significant. The principal component analysis (PCA) was conducted by using SIMCA version 14.0 (Umetrics, Umea, Sweden).

## Results

### Physiochemical Characteristics of Juice Sacs in Different Granulation Processes

**FIGURE 1:**
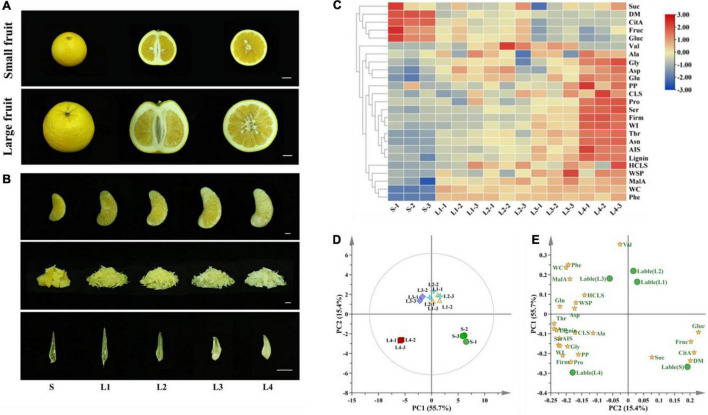
Appearance and physiochemical characteristics of Huyou juice sacs in different granulation degrees. **(A)** Appearance of small-size and large-size fruit. Bar = 2 cm. **(B)** Appearance of juice sacs. Bar = 1 cm. **(C)** Heatmap presenting the variation of physiochemical indexes. **(D)** PCA analysis of samples based on the physiochemical indexes. **(E)** Scores plot of different physiochemical indexes. S, juice sacs from small-size fruit. L1–L4, juice sacs from large-size fruit, which were classified according to the granulation degree. WC, water content; MalA, malic acid; Phe, phenylalanine; HCLS, hemicellulose; WSP, water-soluble pectin; Glu, glutamic acid; Asp, aspartic acid; Thr, threonine; Asn, asparagine; CLS, cellulose; Ala, alanine; Ser, serine; AIS, alcohol insoluble solids; Gly, glycine; PP, protopectin; WI, whiteness index; Pro, proline; Firm, firmness; Suc, sucrose; Fruc, fructose; Gluc, glucose; CitA, citric acid; DM, dry matter.

The fruit size varied greatly among Huyou fruit, even those from the same tree. The small-size and large-size fruit exhibited distinct internal and external qualities (Figure 1A). The large-size fruit tended to have a rough appearance, thick peel, and a large number of seeds, and thus a lower edible rate compared to the small-size fruit. The granulation index was significantly higher in the large-size fruit (2.2) than that in the small-size fruit (0.33) (Supplementary Figure 1). In fact, even the healthy juice sacs from the large-size fruit were more loosely connected compared to those from the small-size fruit (Figure 1B).

The physiochemical quality changes of juice sacs related to the granulation processes were determined. The juice sac hardness increased with the granulation level, with a firmness value of 2.11 N in the L4, which was 12 folds that of S (0.17 N). As the granulated juice sac turned opaque and white gradually, we adopted the WI to reflect the transparency of juice sacs. The WI of the juice sacs ranged from 30.62 to 54.44 and increased with the granulation level (Supplementary Figure 2). The water content of different juice sacs ranged from 86.92 to 89.64%, and no significant difference was found between the different groups (*p* < 0.05). The content of dry matter was slightly higher in the juice sacs from the small-size fruit (13.08%) than that from the large-size fruit (10.36–10.75%) (Supplementary Figure 3). A total of three soluble sugars, two organic acids, and 10 amino acids were identified in the juice sacs of Huyou by GC-MS analysis. Generally, the total contents of soluble sugars and organic acids tended to decrease with the increase in granulation level. The fructose and glucose contents were significantly higher in S than that in L3 and L4. However, the sucrose content was similar among the juice sacs of different granulation levels. Citric acid and malic acid were the dominant organic acids. We found that the citric acid content was significantly higher in the juice sacs from the small-size fruit, while the malic acid content was higher in the juice sacs from the large-size fruit (Supplementary Figure 4). Most of the amino acid contents showed an upward trend with the increase in granulation level. Aspartic acid and asparagine were the dominant amino acids of Huyou juice sac. Notably, the content of the asparagine was significantly higher in the large-size fruit, especially in the L4, which was nearly 17 folds that of the S. Phenylalanine was not detected in the S, but remained steady from L1 to L4 (Supplementary Table 2).

The AIS is composed mainly of the cell wall components, and is constantly increased with the granulation level. The L4 juice sac had an AIS content of 301.76 mg/g DW, which was almost threefold that of S (121.12 mg/g) DW. The protopectin, cellulose, hemicellulose, and lignin contents increased with the granulation level, while the water-soluble pectin increased in the early stage of granulation but decreased in the L4 (Supplementary Figure 5). The loading score of PCA analysis indicated that the firmness, WI, AIS, cellulose, protopectin, lignin, waste soluble pectin, hemicellulose, and several amino acids contents were located on the same side as L3 and L4, suggesting a close correlation of these indexes with juice sac granulation (Figures 1C–E).

### Dynamics of Accumulation and Distribution of Cell Wall Components in the Granulation

**FIGURE 2:**
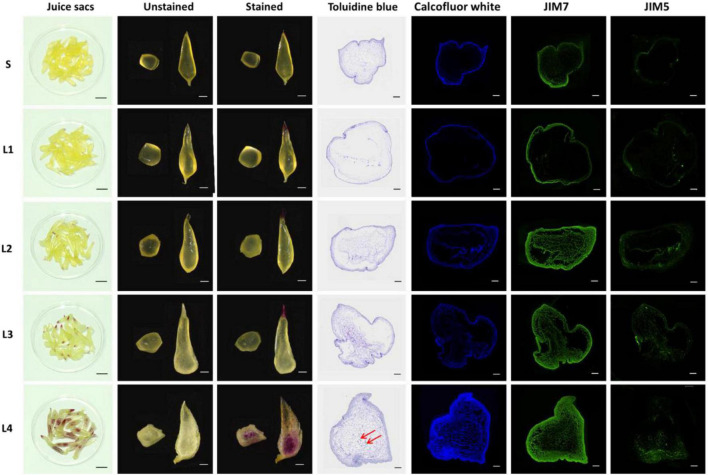
The distribution of cell wall components in the juice sacs. In the first columns, staining of whole juice sacs by phloroglucinol. Bar = 2 cm. In the second and third columns, the transection and longitudinal section of juice sacs are unstained or stained with phloroglucinol. Bar = 1 cm. In the fourth to the seventh columns, labeling of paraffin sections by toluidine blue, calcofluor white, JIM7, and JIM5, respectively. Bar = 500 μm. S, juice sacs from small-size fruit. L1–L4, juice sacs from large-size fruit, which were classified according to the granulation degree. Red arrows showed the cells with lignin deposition.

The dynamics of accumulation and distribution of cell wall components were investigated by chemical or immunofluorescence staining. By phloroglucinol staining of the whole juice sac, the lignin marked in purple-red can be found in the tip of several L2 juice sacs and almost all the L3 juice sacs, as well as in the whole tissue of the L4 juice sacs. The degree of staining increased with the granulation level. For the paraffin section of the juice sac transverse section, toluidine blue-stained lignin was only visible in the cells located in the center of L4 juice sacs. The accumulation dynamics of cellulose were evaluated by calcofluor white labeling, which showed blue fluorescence signal. The cellulose accumulated in both the outer layer and the inner layer cells of the juice sac with the development of granulation. In the L4, the cellulose accumulated significantly, especially in the margin of the juice sac. The pectin distribution was determined by immunolabeling with JIM5 and JIM7, respectively. The JIM7 binds to a relatively heavily methylesterified epitope of galacturonan, while the JIM5 binds to a sparsely methylated homogalacturonan epitope in the cell wall. The signal intensity of JIM7 was significantly higher than that of JIM5 in each group, suggesting that the high-methylesterified pectin was the dominant pectin. Both the signal of JIM5 and JIM7 increased with the granulation, suggesting a dramatically increasing accumulation of pectin in the granulation process, especially the high-methylesterified pectin (Figure 2).

### Phytohormones Metabolites Profiling

**FIGURE 3:**
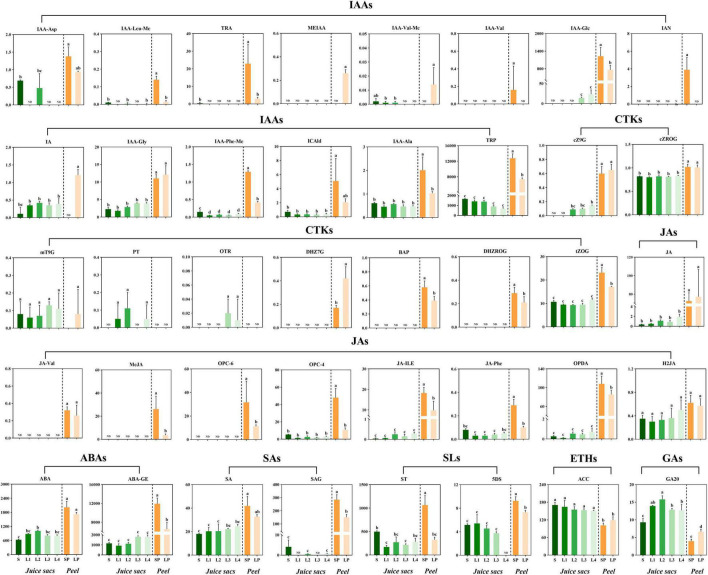
The profiling of phytohormones in the juice sacs and peel of small-size and large-size fruit. Eight classes of phytohormones and/or their metabolites were detected: auxins, cytokinins (CTKs), jasmonic acids (JAs), abscisic acids (ABAs), salicylic acids (SAs), strigolactones (SLs), ethylene (ETHs), gibberellic acids (GAs). S, juice sacs from small-size fruit. L1–L4, juice sacs from large-size fruit, which were classified according to the granulation level. SP, small-size fruit peel. LP, large-size fruit peel. The contents of phytohormones were expressed as ng/g in a fresh weight basis. Data were represented as mean ± standard deviation of three replicates (*n* = 3), with 15 fruit in each replicate. Different lowercase letters in each column represented a significant difference at *p* < 0.05 level by Tukey testing.

Several previous studies had indicated the involvement of phytohormones in the granulation of citrus juice sacs. To explore the correlation of phytohormones with the juice sac granulation, we characterized the phytohormones profiles by LC-MS/MS. A total of 40 phytohormones metabolites were identified in the peel and pulp of Huyou, including auxins, abscisic acids, cytokinins, gibberellin acids (GAs), jasmonic acids (JAs), salicylic acids (SAs), strigolactones (SLs) and/or their intermediate metabolites (Figure 3 and Supplementary Table 3). Most of the phytohormone components were significantly higher in the peel than that in the juice sac, and their profiles in the peel were significantly different between the large-size fruit and the small-size fruit. The peel of small-size fruit contained higher ABA and its derivate ABA-glucosyl ester (ABA-GE) contents than that of the large-size fruit, indicating more active ABA metabolism. The peel of small-size fruit also had a more active IAA metabolism, reflected by the higher contents of 3-indoleacrylic acid (IA), and the synthetic precursors of IAA [L-tryptophan (TRP), tryptamine (TRA), and 3-indoleacetonitrile (IAN)], and the catabolite of IAA [indole-3-acetyl-L-aspartic acid (IAA-Asp)], as well as the storage forms of IAA [methyl indole-3-acetate (MEIAA), indole-3-acetyl-L-valine methyl ester (IAA-Val-Me), 1-*O*-indol-3-ylacetylglucose (IAA-Glc), indole-3-acetyl glycine (IAA-Gly), indole-3-acetyl-L-phenylalanin methyl ester (IAA-Phe-Me), and *N*-(3-indolylacetyl)-L-alanine (IAA-Ala)]. In addition, the peel of small-size fruit contained higher SA and its storage form (salicylic acid 2-glucoside, SAG), as well as higher JA and its synthetic precursors [*cis*(+)-12-oxophytodienoic acid (OPDA), 3-oxo-2-(2-(Z)-pentenyl) cyclopentane-1-hexanoic acid (OPC-6), 3-oxo-2-(2-(*Z*)-pentenyl) cyclopentane-1-butyric acid (OPC-4)], as well as its metabolites [methyl jasmonate (MeJA), *N*-[(–)-jasmonoyl]-L-valine (JA-Val), jasmonoyl-L-isoleucine (JA-Ile), *N*-[(–)-jasmonoyl]-L-phenalanine (JA-Phe), dihydrojasmonic acid (H2JA)]. The peel of small-size fruit also contained higher levels of cytokinins [*cis*-zeatin-9-glucoside (cZ9G), *cis*-zeatin-glucoside riboside (cZROG), meta-topolin-9-glucoside (mT9G), dihydrozeatin-7-glucoside (DHZ7G), 6-benzyladenine (BAP), dihydrozeatin-glucoside riboside (DHZROG) and trans-zeatin-glucoside (tZOG)] (Figure 3). These results indicated more active phytohormone metabolism in the peel of small-size fruit.

For the juice sacs of different granulation levels, six compounds related to JA metabolism were identified, including OPDA, OPC-4, JA, H2JA, JA-Ile, and JA-Phe, which were increased with the granulation level. SA content was also increased with the granulation level and peaked in the L4 stage. The cytokinins cZ9G, cZROG, mT9G, ortho-topolin riboside (oTR), para-topolin (pT), and tZOG were elevated with the granulation. Among them, the oTR was detectable only in L3 and L4, and the pT was detectable only in L1, L2, and L4 juice sacs. Four storage forms of auxins (IAA-Glc, IAA-Gly, and IAA-Phe-Me) also showed a mild increase from L1 to L4, while the IA and IAA-Ala showed no significant change, and the catabolite of IAA (IAA-Asp) was only detectable in the S and L2 (Figure 3). The ABA content increased in the L1 and L2 stages, while decreased in the L3 and L4 stages. ABA-GE showed a significant increase in the L3 and L4 stages.

### RNA-Seq Data Revealed Differentially Expressed Genes

**FIGURE 4:**
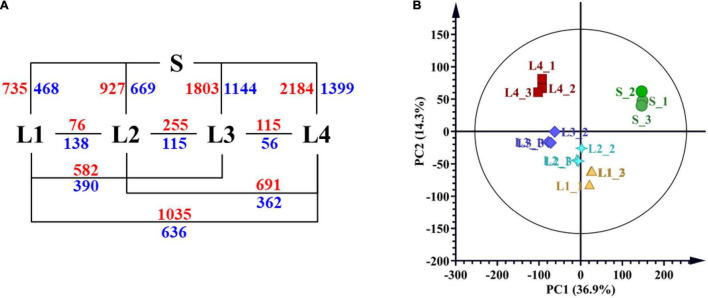
RNA-seq results of different juice sacs. **(A)** Numbers of differentially expressed genes (DEGs) from the pairwise comparison of different juice sacs. **(B)** PCA analysis presenting the difference of RNA-seq results of different samples. S, juice sacs from small-size fruit. L1–L4, juice sacs from large-size fruit, which were classified according to the granulation degree.

We carried out RNA-seq analysis to get more understanding of the biological rules of juice sac granulation. The RNA-seq generated 135.26 Gb of clean data for five groups of juice sacs with three biological replicates in each group (Supplementary Table 4). The above-mentioned transcript dataset was used to identify DEGs. Pairwise comparisons of gene expression levels were conducted to identify the DEGs that met the criteria FDR ≤ 0.05 and | log2 fold change| >1 in each pairwise comparison. A total of 4,429 DEGs were found, accounting for 18.24% of the total annotated genes. The number of DEGs ranged from 171 to 3583 among comparisons and increased with the interval of granulation level (Supplementary Table 5 and Figure 4). Ten of the genes were selected for the RT-qPCR verification, and the relative expression level of these candidate genes was coincidental with RNA-seq, with a coefficient of determinant *R*^2^ of 0.7305 (Supplementary Figure 6 and Supplementary Table 8).

#### Analysis of Differentially Expressed Genes Expression Trends by Mfuzz Clustering

**FIGURE 5:**
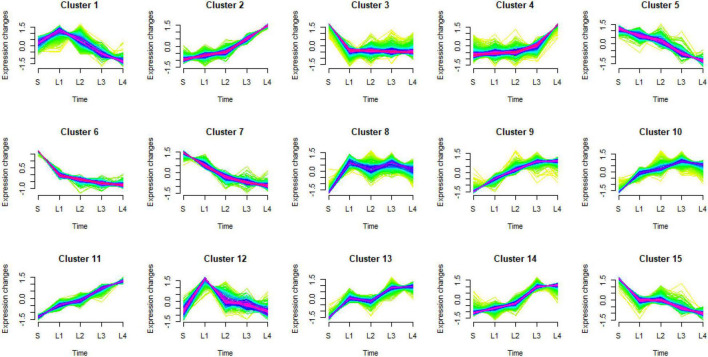
Results of Mfuzz clustering of 4,429 differentially expressed genes (DEGs). S, juice sacs from small-size fruit. L1–L4, juice sacs from large-size fruit, which were classified according to the granulation degree.

**FIGURE 6:**
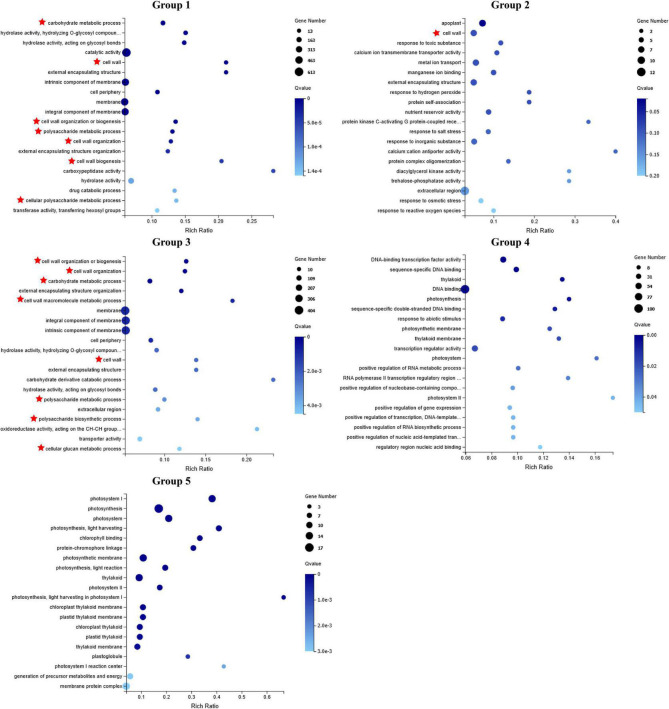
Gene Ontology (GO) enrichment of selected category of differentially expressed genes (DEGs) based on Mfuzz cluster analysis.

Mfuzz clustering analysis was carried out to investigate how the DEGs responded to the granulation. All 4,429 DEGs were grouped into 15 clusters. Clusters 2, 9, 11, and 14 showed a similar tendency with the granulation level (group 1), clusters 5, 6, 7, and 15 showed the opposite tendency with the granulation level (group 4). Clusters 1 and 12 had higher expression levels in the juice sacs from large-size fruit than that from small-size fruit, while decreased with the development of granulation (group 2). Clusters 4, 8, 10, and 13 increased at a certain granulation stage (group 3). Cluster 3 had lower expression in the juice sacs from the large-size fruit than that from the small-size fruit but remained stable in different stages of granulation (group 5) (Figure 5 and Table 1). These indicated that group 1 to group 3 might be positively correlated to the granulation level, while group 4 and group 5 might be negatively correlated to the granulation level. To further analyze the potential function of these DEGs, the GO enrichment analysis was carried out. Genes related to the cell wall organization and biogenesis, cell wall macromolecule metabolic process, carbohydrate metabolic process, and the polysaccharide metabolic process were significantly enriched in groups 1, group 2, and group 3, corresponding to the increase of cell wall components in the granulated juice sac (Figure 6 and Supplementary Table 6).

**TABLE 1:** Granulation responsive category analysis of DEGs based on Mfuzz cluster analysis.

Group	Response	Clusters (numbers of DEGs)
Group 1	Increased with the granulation	2(378), 9(308), 11(312), 14(406)
Group 2	Increased in the large-size fruit, while decreased with the development of granulation	1(190), 12(157)
Group 3	Increased at a certain stage of the granulation	4(343), 8(230), 10(290), 13(326)
Group 4	Decreased with the granulation	5(227), 6(333), 7(280), 15(311)
Group 5	Decreased compared to small-size fruit but remained stable in different stages of granulation in the large-size fruit	3(338)

#### Analysis of Co-expression Networks Revealed Granulation Related Genes by Weighted Gene Co-expression Network Analysis

**FIGURE 7:**
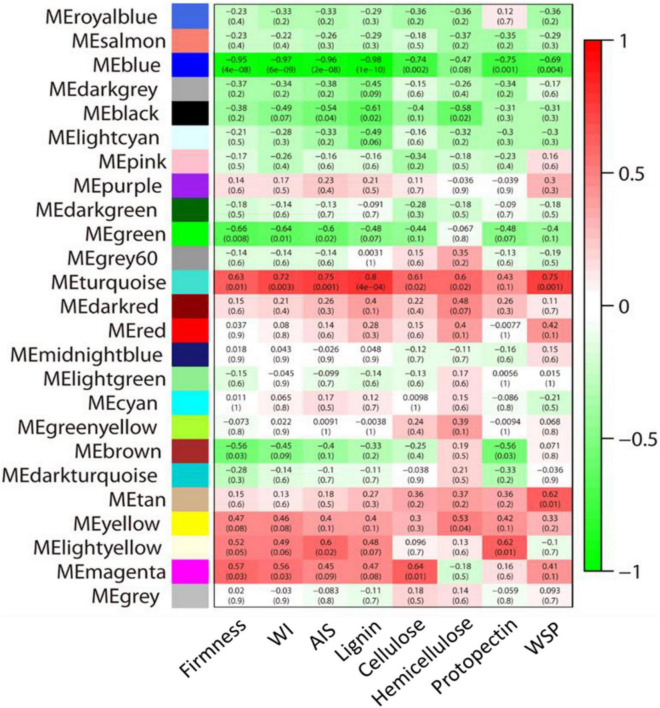
Module-trait relationship analysis by WGCNA. Numbers in the squares presented the module-trait correlations (with corresponding *p*-values in parentheses). The color scale represents the module-trait correlations from –1 (green) to 1 (red). Panels WI, AIS, and WSP represent the whiteness index, alcohol insoluble solids, and water-soluble pectin, respectively.

To get more understanding of the genes that responded to the granulation, the WGCNA analysis of all genes was performed. We performed a module-trait relationship analysis using AIS contents, water-soluble pectin contents, protopectin contents, cellulose contents, hemicellulose contents, lignin contents, firmness, and hunter value as trait data. A total of 25 modules were identified. The module-trait relationships revealed that the “turquoise” module was highly positively correlated with all the cell wall contents, and the “blue” module was highly negatively correlated with the cell wall contents (Figure 7 and Supplementary Table 7).

#### Candidate Differentially Expressed Genes Set Construction Based on the Mfuzz and Co-expression Analysis

The candidate DEGs set was constructed by combining the results of Mfuzz analysis and co-expression analysis. The turquoise module shared 778 DEGs with group 1, 11 DEGs with group 2, and 569 DEGs with group 3 of Mfuzz analysis respectively. These DEGs were selected as the candidate genes that were positively related to the granulation processes (DEGs set 1). The blue module shared 351 DEGs with group 4, and 28 DEGs with group 5 of Mfuzz analysis respectively. These DEGs were selected as the candidate genes that negatively related to the granulation processes (DEGs set 2).

In the DEGs set 1, 31 DEGs related to the cell wall and the polysaccharides metabolism were found. The β-1,4-xylosyltransferase IRX9, cellulose synthase, xyloglucan: xyloglucosyl transferase, xyloglucan galactosyltransferase (MUR3), and α-1,4-galacturonosyltransferase that required for the synthesis of cellulose, pectin, and other cell wall polysaccharides were positively correlated with the granulation process. Several transcripts of expansin, which is involved in the elongation of cells, were up-regulated in the granulation. Except for the genes related to the cell wall polysaccharides biosynthesis, a certain number of genes related to the pectin and cellulose degradation were also found in the DEGs set 1 and DEGs set 2, including the genes encoding polygalacturonase, pectinesterase, β-glucosidase, β-galactosidase, endo-1,3(4)-β-glucanase, endoglucanase, and pectate lyase. In addition, eight transcripts of peroxidase, which might be involved in the lignin metabolism, were up-regulated (Table 2).

**FIGURE 8:**
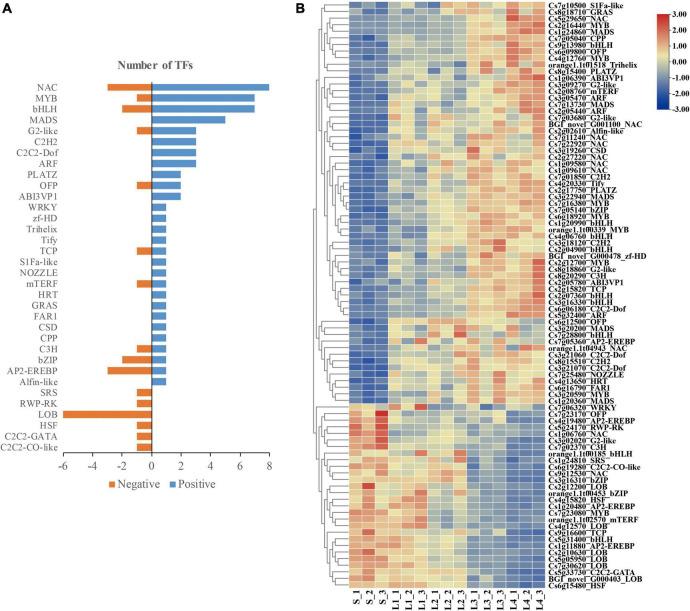
Transcription factors (TFs) correlated to the granulation process. **(A)** Number of TFs in different families. **(B)** Heatmap presenting the expression patterns of different TFs in response to granulation process.

In the selected DEGs, a total of 89 TFs were found to be related to the granulation process. Among them, 62 TFs belonging to 28 TFs families were found in the DEGs set 1, with the NAC (8), MYB (7), bHLH (7), and MADS (5) as the top abundant families; 27 TFs belonging to 16 TFs families were found in the DEGs set 2, with the LOB (6) as the top abundant family (Figure 8A). The expression pattern of the selected TFs transcripts was correlated to the granulation processes of juice sacs (Figure 8B).

## Discussion

As one of the most common physiological disorders that happened in citrus fruit, juice sac granulation had many adverse effects on fruit quality, which had been a concern by the producer and researchers for a long time. The granulated juice sacs have increased firmness (Chen et al., 2021), lower soluble sugars and organic acids contents (Shomer et al., 1989; Sharma et al., 2006; Wang et al., 2014), while higher cell wall components contents, including pectin, cellulose, and lignin (Yao et al., 2018; Cao et al., 2020; Shi et al., 2020). In addition, the main volatile compounds, such as limonene and β-myrcene, were dramatically decreased in the granulated juice sac (Cao et al., 2020; Yao et al., 2020). This indicated a global change in both the primary and secondary metabolisms.

In the present study, we found a significant accumulation of amino acids in the granulated juice sac of Huyou fruit. All of the 10 detected amino acids showed a trend of increase, especially asparagine (Supplementary Table 2). In previous studies, amino acid accumulation is found to be involved in the physiological responses of plants to natural senescence or adverse environments. Eg., Herrera-Rodríguez et al. (2006) found that the increase of asparagine was correlated to the natural senescence of cotyledon in sunflower. Downs et al. (1997) reported that the postharvest senescence of broccoli can be delayed by cytokinin treatment, and meanwhile, the increase of asparagine and glutamine content was delayed. Thus, the increase of amino acids in our study provided a hint that there might be some correlation between the juice sac granulation and the adverse environment response and senescence of citrus fruit. But the further study was required to prove this.

The cell wall component accumulation is another obvious change in granulation. It directly led to the texture changes of granulated juice sac. Thus, it is the most commonly reported phenomenon in previous studies. Yao et al. (2018) deduced that the soluble sugars and organic acids were degraded, and the metabolic direction changed to the synthesis of cell wall compounds, such as cellulose and pectin during the granulation process, based on the results of RNA-seq analysis of juice sacs in different granulation level.

Lignin is reported to be the chief compound that contributes to the cell wall changes in the granulated juice sac of pomelo fruit (Shi et al., 2020). According to the toluidine blue and phloroglucinol staining results from Shi et al. (2020), lignin was abundantly distributed on the margin of the juice sacs in Huanong red-fleshed pomelo. In the present study, we found that the accumulation of lignin in Huyou can be detected only after the L2 stage, and its early accumulation happened at the tip of the juice sac. The massive accumulation of lignin can be seen only in the late stage of granulation (L4). This was quite different from the reported results for pomelo fruit. Thus, the lignin might not be the chief contributor to the texture changes of Huyou in the early granulation stage.

Pectin and cellulose contents are also reported to be increased in the granulated juice sac of different citrus fruit (Sinclair and Jolliffe, 1960; Yao et al., 2018). The structural changes of pectin had been reported in the previous study of Sinclair and Jolliffe (1960), with the methylation level of the pectin increased in the granulated juice sac. In our study, the low-esterified and high-esterified pectin was immunolabeled by JIM5 and JIM7, respectively. We found that the JIM7 labeling signal increased earlier and more quickly than the JIM5 labeling signal in the granulation process, suggesting the occurrence of pectin methylesterification in the granulation process. This result is consistent with the results of Sinclair and Jolliffe (1960).

**TABLE 2:** Selected DEGs related to the cell wall formation.

	Functional annotation	Change pattern
Cs6g04870	Beta-1,4-xylosyltransferase IRX9	P
Cs6g06530	Expansin	P
Cs6g21460	Expansin	P
Cs8g18640	Expansin	P
Cs5g07854	Expansin	P
BGI_novel_G000701	Expansin	P
Cs8g01350	Expansin	P
orange1.1t01658	Xylan 1,4-beta-xylosidase	P
Cs8g08680	Xylan 1,4-beta-xylosidase	P
Cs9g02570	Xylan 1,4-beta-xylosidase	P
Cs5g29200	Cellulose synthase A	P
Cs1g03980	Cellulose synthase A	P
Cs2g04590	Cellulose synthase A	P
Cs5g01960	Cellulose synthase A	P
Cs5g01970	Cellulose synthase A	P
Cs4g08560	Cellulose synthase A	P
Cs7g01740	Cellulose synthase A	P
Cs7g05150	Cellulose synthase A	P
Cs8g01130	Cellulose synthase	P
Cs7g19940	Callose synthase	P
orange1.1t02029	Callose synthase	P
Cs1g21130	Xyloglucan:xyloglucosyl transferase	P
Cs2g22200	Xyloglucan:xyloglucosyl transferase	P
orange1.1t02385	Xyloglucan:xyloglucosyl transferase	P
Cs8g15720	Xyloglucan:xyloglucosyl transferase	P
orange1.1t01879	Xyloglucan galactosyltransferase (MUR3)	P
Cs9g17930	Xyloglucan galactosyltransferase (MUR3)	N
orange1.1t01879	Alpha-1,4-galacturonosyltransferase	P
Cs6g04560	Peroxidase	P
Cs8g10400	Peroxidase	N
Cs1g20230	Peroxidase	P
Cs2g03110	Peroxidase	P
Cs5g32270	Peroxidase	P
Cs8g14410	Peroxidase	P
orange1.1t02046	Peroxidase	P
Cs6g20170	Peroxidase	P
orange1.1t02044	Peroxidase	P
orange1.1t01518	Pectinesterase	P
Cs1g16550	Pectinesterase	P
Cs5g33420	Pectinesterase	P
Cs9g14450	Pectinesterase	P
Cs2g19350	Pectinesterase	P
BGI_novel_G000496	Pectinesterase	P
Cs4g06630	Pectinesterase	P
Cs9g14330	Pectinesterase	P
Cs3g14610	Pectinesterase	N
Cs4g06890	Pectinesterase	N
BGI_novel_G000487	Beta-glucosidase	P
Cs4g11970	Beta-glucosidase	P
Cs2g04190	Beta-glucosidase	P
Cs3g26470	Beta-glucosidase	P
Cs6g15160	Beta-glucosidase	P
Cs8g03320	Beta-glucosidase	P
Cs8g03370	Beta-glucosidase	P
BGI_novel_G001016	Beta-glucosidase	P
Cs3g23360	Beta-glucosidase	P
Cs7g11210	Beta-glucosidase	P
Cs2g11040	Beta-glucosidase	N
Cs8g08270	Beta-galactosidase	P
Cs3g18120	Beta-galactosidase	P
Cs1g08380	Beta-galactosidase	P
Cs1g24730	Beta-galactosidase	P
Cs4g14090	Beta-galactosidase	P
Cs4g07080	Beta-galactosidase	N
Cs5g03170	Polygalacturonase	P
orange1.1t01777	Polygalacturonase	P
Cs6g07040	Endo-1,3(4)-beta-glucanase	P
Cs6g11570	Endo-1,3(4)-beta-glucanase	P
Cs6g19060	Endo-1,3(4)-beta-glucanase	P
Cs5g03000	Endoglucanase	P
Cs5g33760	Endoglucanase	P
Cs5g02320	Endoglucanase	P
Cs1g21260	Endoglucanase	N
Cs1g03670	Pectate lyase	P
Cs7g21940	Pectate lyase	P

*P, positively correlated to granulation; N, negatively correlated to granulation.*

The juice sac granulation is a complex physiologic disorder that might be caused by a series of factors, such as genetic background (El-Zeftawi et al., 1985; Sharma et al., 2006), cultivation management (Shiraishi et al., 1999; Ritenour et al., 2004), fruit size (Kang et al., 2022), and harvest maturity (Burns and Albrigo, 1998). Most of these factors might be related to the phytohormones changes. Several studies provided pieces of evidence. Wang et al. (1997) found that the ABA content of section drying fruit was significantly higher than that of the normal fruit, whereas the IAA and cytokinin contents were lower in section drying fruit, suggesting that the change of phytohormones balance might be related to the granulation. Shi et al. (2020) found that exogenous ABA treatment significantly accelerated the *in vitro* cultivated juice sac granulation while IAA effectively inhibited this process, which gave direct evidence of the role of phytohormones in the juice sac granulation. In the present study, we found more complex rules on the phytohormones changes. The ABA content and its catalytic form (ABA-GE) (Burla et al., 2013) increased in the granulated juice sac. The ABA-GE had been found to be correlated to drought, osmotic stress, and other abiotic stresses (Sauter et al., 2002). The auxins seem to exist in storage form in Huyou fruit. Three storage forms of IAA increased with granulation, which was different from the result of Wang et al. (1997). In addition to auxins, ABA, and cytokinins that had been reported previously, the resistance-related phytohormones JA and SA were positively correlated to the granulation. In *Arabidopsis thaliana*, JA promoted the expression of cell wall-modifying enzymes (i.e., pectin methylesterases) in abiotic stress (Bethke et al., 2015). SA was also related to the immune responses in the plant, by strengthening the cell wall by increasing lignin and callose production (Butselaar and Ackerveken, 2020). These results indicated that several phytohormone families were related to the juice sac granulation. In addition, we also noticed that the phytohormones contents varied significantly between the peel and the pulp. We assumed that there might be two reasons for this phenomenon. First, the peel had a much lower water content and higher cell number compared to the juice sac, which can at least partially explain the differences. Second, the life activity in the citrus peel might be more exuberant than the juice sac in the Huyou fruit, just as is reported in the loose skin citrus in the study of Ding et al. (2015), in which the direction of material transport in the postharvest citrus fruit was reported to be from the juice sac to the peel. The effects of the phytohormones distribution on granulation are worth an in-depth study. As the granulated syndrome is perceptually invisible from the appearance, and the regulatory factors are uncertain, the research on its underlying mechanism relies on special models. Shi et al. (2020) studied the regulatory mechanisms of granulation by using *in vitro* cultivated juice sacs. Our previous study on the relationship between fruit size and granulation rate indicated that the large-size fruit could be used as material for granulation research (Kang et al., 2022). By transcriptome comparison of the normal juice sac and granulated juice sac of Ponkan citrus, Yao et al. (2018) found that the genes encoding cinnamyl alcohol dehydrogenase (CAD) in the lignin synthesis pathway, UDP-glucose pyrophosphorylase (UGP) involved in the cellulose synthesis, and galacturonosyltransferase (GAUT) involved in the pectin synthesis were up-regulated in the granulated juice sac of orange, while the genes encoding pectin methylesterase (PME) involved in the hydrolysis of pectin, and β-D-xylosidase (BXL) involved in the degradation of xylan, were down-regulated in the granulated tissue. In another transcriptome comparison of the normal juice sac and granulated juice sac of orange fruit, Yao et al. (2020) screened out UDP-glucuronate 4-epimerase (GAE) and GAUT in pectin biosynthesis, cellulose synthase complex (CSC) in cellulose synthesis, and key genes in the phenylalanine metabolic pathway were up-regulated in the granulated juice sac. Wu et al. (2020) reported the up-regulation of genes encoding phenylalanine ammonia-lyase (PAL), CAD, and peroxidase involved in lignin synthesis, and the down-regulation of polygalacturonase involved in pectin degradation and cellulase (CL) involved in cellulose degradation. In the granulated juice sac of pomelo fruit, in which the lignin was excessively over-accumulated, critical genes in the phenylalanine metabolism and lignin biosynthesis pathway showed different expression patterns. The *PAL1*, *PAL2*, *PAL5*, *LAC11*, *CCoAOMT3*, and *CCoAOMT4* are highly expressed at the very beginning, *4CL*, *C3H*, *LAC4*, *LAC5*, *LAC7*, *LAC13*, *LAC15*, and *LAC22* were up-regulated in the late stage of granulation (Shi et al., 2020). In our study on Huyou, combining Mfuzz trend analysis and WGCNA analysis, a series of genes involved in the cell wall formation and modification was found to be correlated with the granulation process, including the genes encoding β-1,4-xylosyltransferase IRX9, cellulose synthase, xyloglucan: xyloglucosyl transferase, xyloglucan galactosyltransferase MUR3, and α-1,4-galacturonosyltransferase, which had been reported to be required for the synthesis of cellulose, pectin, xylan, and other cell wall polysaccharides (Jensen et al., 2011). The expansin, which is involved in the elongation of cells (Cho and Kende, 1997), was up-regulated in the granulation. Except for the genes related to the cell wall polysaccharides biosynthesis, a certain number of genes related to pectin and cellulose degradation were also found to be up- or down-regulated, including the polygalacturonase, pectinesterase, β-glucosidase, β-galactosidase, endo-1,3(4)-β-glucanase, endoglucanase and pectate lyase (Table 2). Combining with the dynamic changes of cell wall polysaccharides contents, in which the cellulose, protopectin, and lignin increased from S to L4, while the water-soluble pectin and hemicellulose contents increased mainly in the early stage of granulation and fluctuated insignificantly during L2 to L4 (Supplementary Figure 6), we assumed that the cell wall remodeling happened in the granulation, involving not only the biosynthesis but also the degradation of some polysaccharides.

The transcriptome comparison between normal and granulated juice sacs also suggested that a series of TFs might be related to the granulation process. Yao et al. (2020) reported the up-regulation of 34 TFs families in the granulated orange juice sac, with the top abundant families of bHLH, WRKY, ERF, MYB, and 46 down-regulated families, with the top abundant families of bHLH, MYB-related, NAC, ERF, bZIP, and LBD. In the Ponkan granulated juice sacs, 17 TFs families were differentially expressed, with LBD being down-regulated, and bHLH, ERF, MYB, and NAC being up-or down-regulated (Yao et al., 2018). Shi et al. (2020) found 24 differentially expressed TFs significantly correlated to the lignin accumulation during the pomelo juice sac granulation, in which the *MYB* accounted for the largest number. In addition, Shi et al. (2020) have proved that *CgMYB58* was critical for the granulation-related lignin accumulation, which modulates a series of key genes in the lignin biosynthesis pathway. Jia et al. (2018, 2019) reported that the *CsMYB330*, *CsMYB308*, and *CsMYB85* regulated the lignification of sweet orange juice sac by regulating the *4CL1* expression. In our study of Huyou, the top abundant TFs families positively related to granulation were found to be the NAC (8), MYB (7), bHLH (7), and MADS (5), while the top abundant TFs family negatively related to granulation was the LOB (6). Our results partially agreed with the former studies. Taken together, it can be deduced that the NAC, MYB, bHLH and LOB might be important in the regulation of granulation.

A series of NAC family members are involved in the biosynthesis of secondary cell walls, with the target genes involved in the biosynthesis of secondary cell wall components, such as cellulose, hemicellulose, and lignin (Nakano et al., 2015). *NAC* genes are induced by at least one type of stress and responded to ABA, JA, and SA (Puranik et al., 2012). MYBs involved in the biosynthesis of phenylpropanoids have been reported to be targeting a vast of genes for secondary cell wall metabolism, including cellulose synthase genes, xylan biosynthetic genes, and a series of genes in the lignin biosynthetic pathway (Nakano et al., 2015); bHLH is involved in JA-modulated secondary metabolism (Goossens et al., 2017). Some components of MADS had been reported to be involved in the ABA signaling and regulated the plant responses against abiotic stress (Castelán-Muñoz et al., 2019). Considering the reported complex interactions among the NAC, MYB, bHLH, MADS, and LOB TFs and the phytohormones ABA, JA, and SA, as well as their responses to the senescence signals, abiotic and biotic stress factors, we can deduce that the juice sac granulation might be related to the stress responses, ripening, and senescence of Huyou fruit. In addition to the reported *CgMYB58*, *CsMYB330*, *CsMYB308*, and *CsMYB85* (Jia et al., 2018, 2019; Shi et al., 2020), more elements and mechanisms of the complex regulatory network in the process of granulation remain to be explored in future studies.

## Conclusion

Huyou fruit of large size tends to develop juice sac granulation. The cell wall component accumulation is one of the most obvious physiochemical changes correlated with the granulation process, with the methylesterified pectin accrued early in the outer layer cells, and lignin accumulated dramatically lately in the inner layer cells of the juice sac. The accumulation of cell wall components led to the firmness increase of the granulated juice sac. Several phytohormone components of auxin, ABA, cytokinin, JA, SA, and/or their storage forms in the juice sacs were positively correlated to the granulation, indicating active and complex phytohormones metabolism in the granulation process. A series of genes related to the cell wall polysaccharides biosynthesis and structural modification was mined out from the DEGs of RNA-Seq data, according to their expression trends and their correlation with the granulation traits. In addition, several TFs families were also mined out, with the NAC, MYB, bHLH, and MADS families being the top abundant TFs positively correlated with the granulation process, while the LOB family was the top abundant TFs negatively correlated with the granulation process. More elements and mechanisms of the complex regulatory network in the process of granulation remained to be explored in future studies.

## Data Availability Statement

The sequencing data have been deposited in the NCBI Sequence Read Archive (https://www.ncbi.nlm.nih.gov/sra/), with the BioProject ID: PRJNA827278.

## Author Contributions

CK contributed to the investigation, data collection and analysis, and writing the original draft. AJ, HY, and GZ contributed to the samples collection. YW contributed to the reviewing and editing. JC contributed to the supervision, data analysis, and writing—review and editing. CS contributed to the supervision, reviewing and editing. All authors have read and agreed to the published version of the manuscript.

## Conflict of Interest

The authors declare that the research was conducted in the absence of any commercial or financial relationships that could be construed as a potential conflict of interest.

## Publisher’s Note

All claims expressed in this article are solely those of the authors and do not necessarily represent those of their affiliated organizations, or those of the publisher, the editors and the reviewers. Any product that may be evaluated in this article, or claim that may be made by its manufacturer, is not guaranteed or endorsed by the publisher.

## Footnotes

1
https://www.ncbi.nlm.nih.gov/assembly/GCF_000317415.1

## Supplementary Material

Supplementary Table 1 Primers used for RT-qPCR.

Supplementary Table 2 The physiochemical indexes of small-size fruit and large-size fruit.

Supplementary Table 3 Phytohormones profiles in juice sacs and peel.

Supplementary Table 4 Details regarding the RNA-seq data.

Supplementary Table 5 Details regarding the full-length sequences.

Supplementary Table 6 Transcripts in different clusters following the Mfuzz analysis.

Supplementary Table 7 Transcripts in MEturquoise and MEblue modules.

Supplementary Table 8 Relative expression level of candidate transcripts for the RT-qPCR validation.
