# Supplementary material for: Integrated Physiochemical, Hormonal, and Transcriptomic Analysis Revealed the Underlying Mechanisms for Granulation in Huyou (Citrus changshanensis) Fruit
Source: Front Plant Sci. 2022 Jul 14;13:923443. doi: 10.3389/fpls.2022.923443 (PMC9330425; doi:10.3389/fpls.2022.923443)
Supplement: Supplementary file 2 [file Data_Sheet_1.docx]

**Supplement Figures**

**Fig. S1 The general fruit quality of small and large Huyou fruit.**

(A) Citrus color index (CCI); (B) Total soluble solids (TSS); (C) Edible rate; (D) Fruit shape index; (E) Granulation index; (F) Seed number. Data were represented as mean ± standard deviation of 30 replicates (n = 30). Statistical significance was calculated by t-test: *, P < 0.05; ns, non-significant.


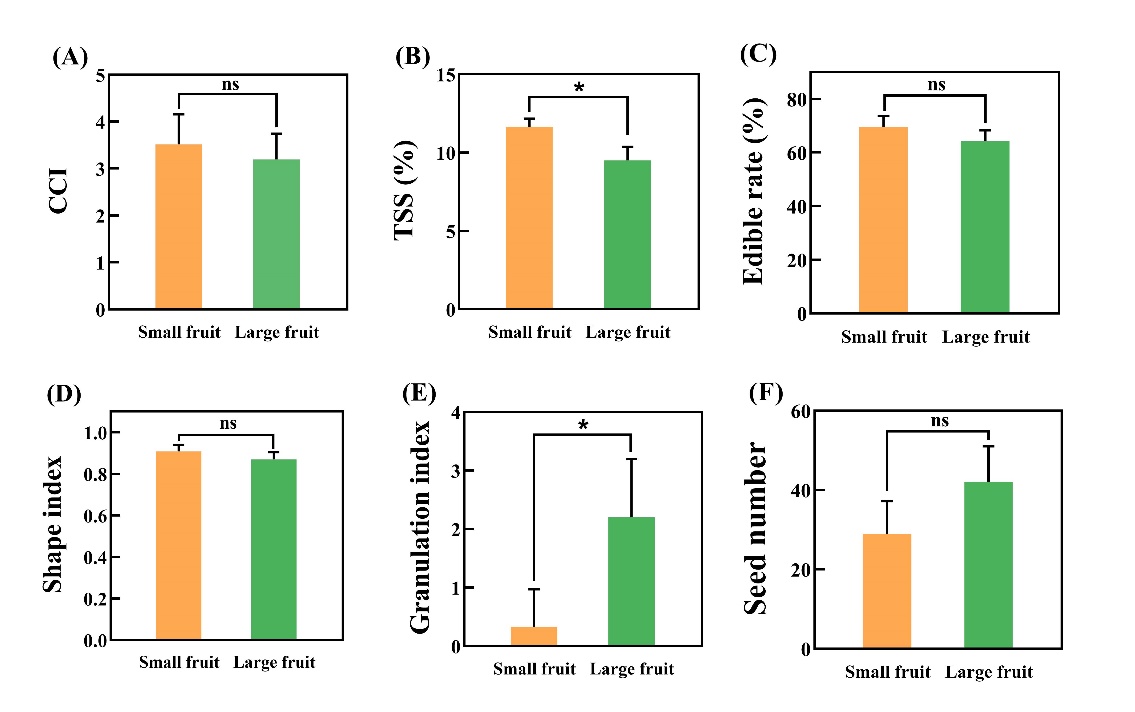


**Fig. S2 Dynamic changes of the firmness and whiteness index (WI) of juice sacs during the granulation process**

(A) Firmness. (B) WI. S, juice sacs from small-size fruit. L1-L4, juice sacs from large-size fruit, which were classified according to granulation level. Data were represented as mean ± standard deviation of 30 replicates (n = 30). Different lowercase letters in the column represented significantly difference at p < 0.05 level by Tukey testing.


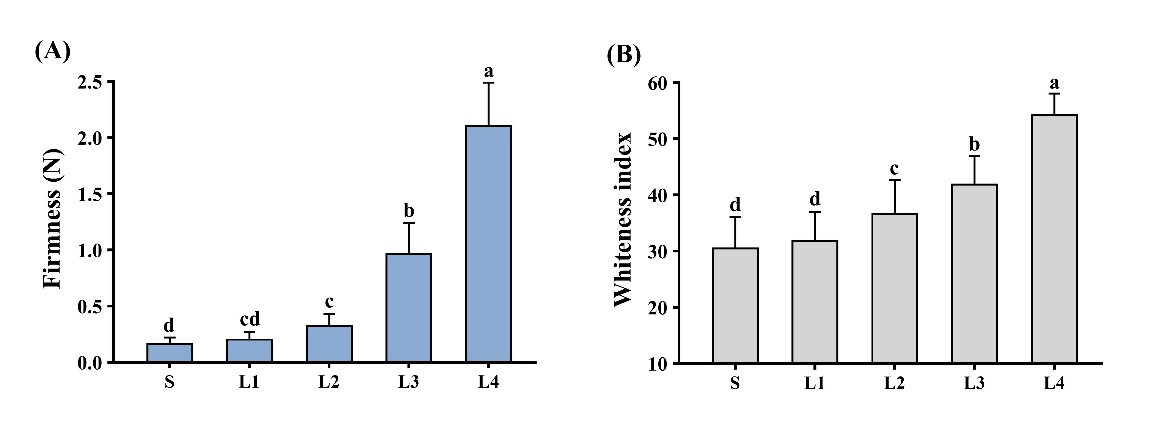


**Fig.S3 Dynamic changes of the water content of juice sacs during the granulation process**


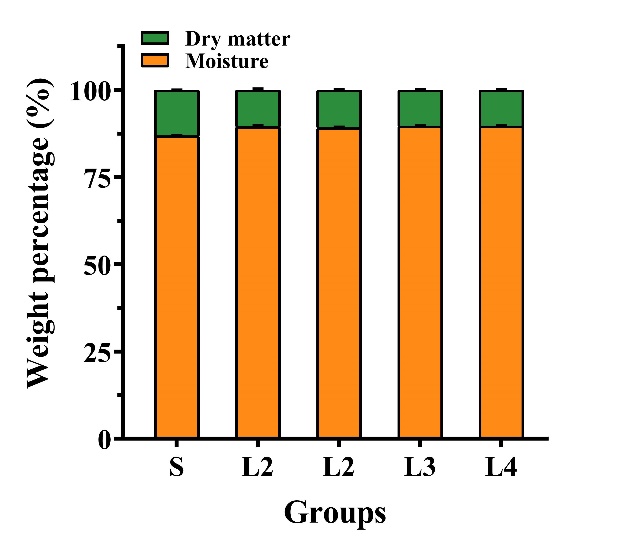


**Fig.S4 Dynamic changes of the soluble sugars (A) and organic acids (B) of juice sacs during the granulation process**

S, juice sacs from small-size fruit. L1-L4, juice sacs from large-size fruit, which were classified according to granulation level. The contents of soluble sugars and organic acids were expressed as g/kg on a dry weight basis. Data were represented as mean ± standard deviation of three replicates (n = 3), with the combination of juice sacs from 15 fruits in each replicate. Different lowercase letters in each column of the same component represented significantly difference between different groups at p < 0.05 level by Tukey testing.


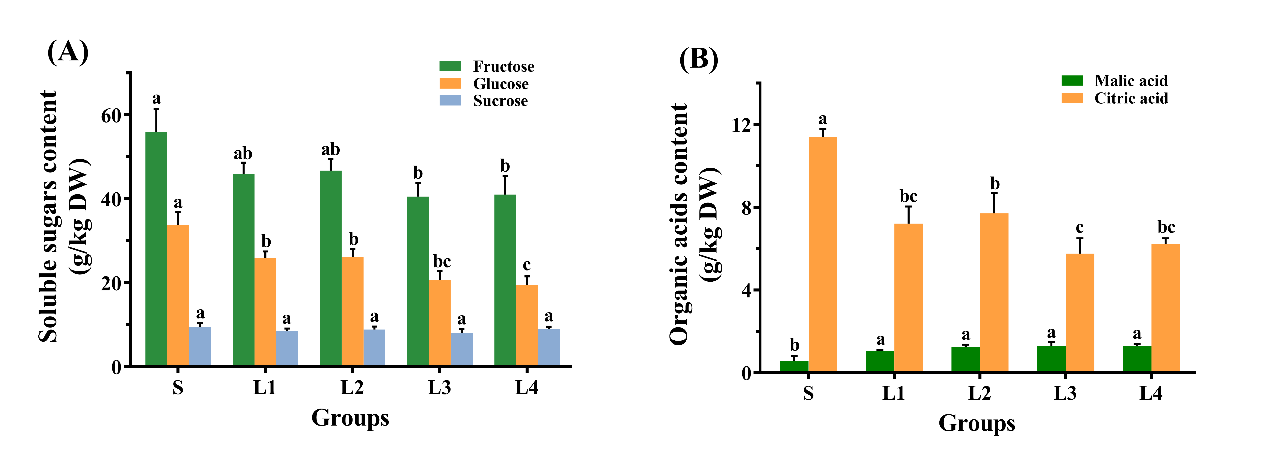


**Fig.S5 Dynamic changes of the cell wall components contents of juice sacs during the granulation process**

(A) Alcohol insoluble solids (AIS) content. (B) Lignin content. (C) Water-soluble pectin content. (D) Protopectin content. (E) Cellulose content. (F) Hemicellulose content. S, juice sacs from small-size fruit. L1-L4, juice sacs from large-size fruit, which were classified according to granulation level. The contents of AIS, water-soluble pectin, protopectin, cellulose and hemicellulose were expressed as mg/g on a dry weight basis; The lignin content was expressed as %ABSL on a dry weight basis. Data were represented as mean ± standard deviation of three replicates (n = 3), with more than 20 fruits in each replicate. Different lowercase letters in the column represented significantly difference at p < 0.05 level by Tukey testing.


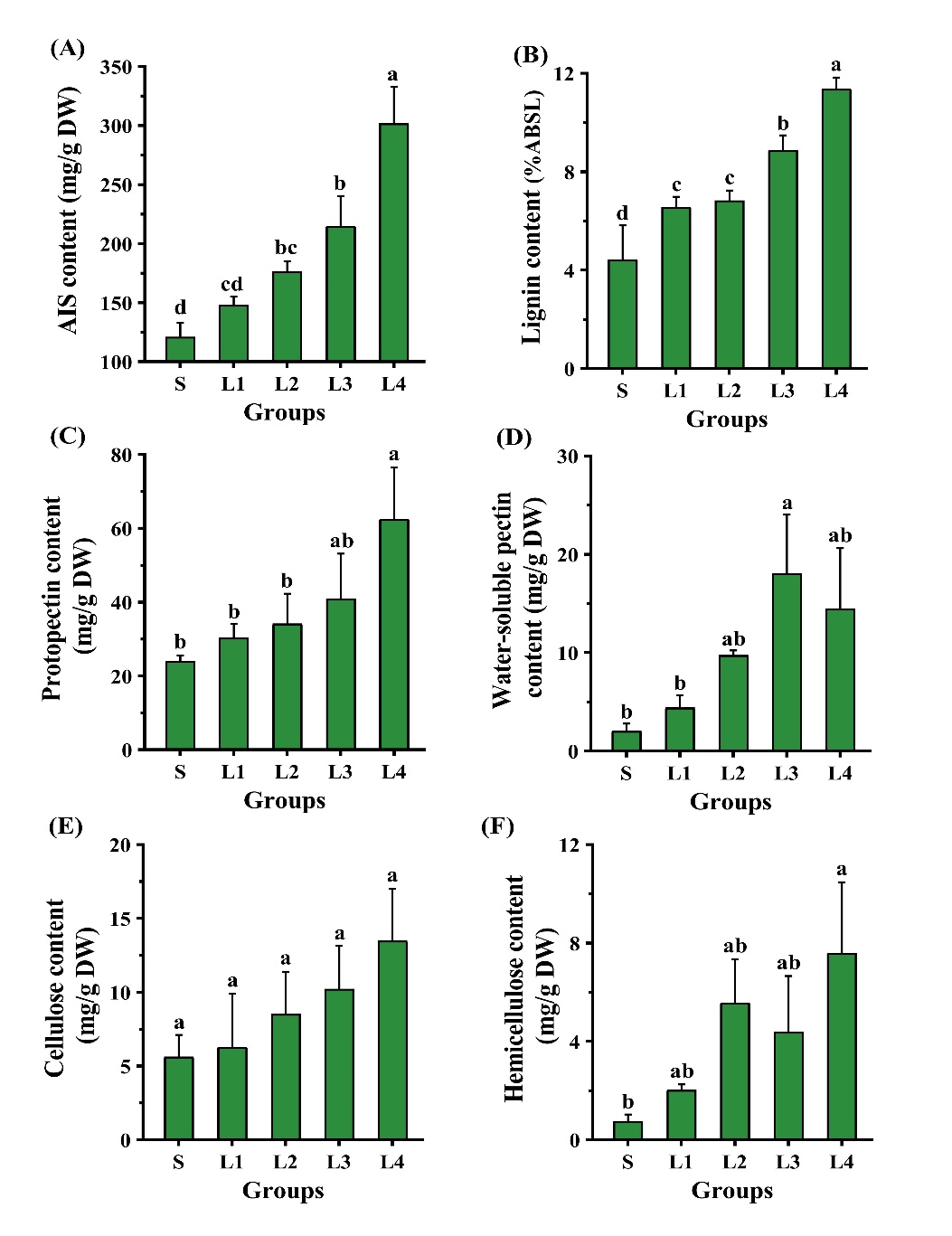


**Fig. S6 Validation of differentially expressed transcripts by RT-qPCR**

Correlation analysis between the result of RNA-Seq and RT-qPCR was performed based on the expression level. X-axis represents the log2FC of FPKM value, Y-axis represents the log2FC of relative expression by RT-qPCR, using the sample of S stage as control.


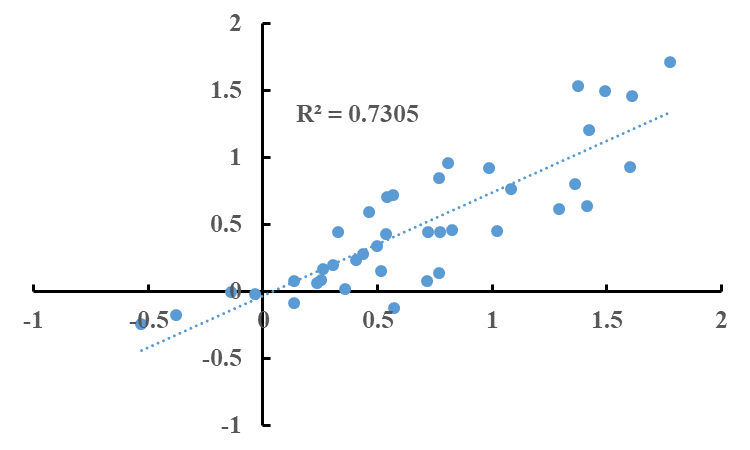


**Method S1**

1.1 General Fruit Quality Analysis

For the general fruit quality analysis, 30 fruit from small- and large-size fruit were randomly selected, respectively. Rind color measurement was conducted using a colorimeter (Hunterlab, USA). Four evenly distributed equatorial sites of each fruit were selected for the determination. The CIE 1976 *L * ɑ * b ** color scale was adopted and citrus color index (CCI) of each fruit was calculated according to the following formula (Cao et al., 2019).

$$\text{CCI =}\frac{\text{a}^{\text{*}}\text{×1000}}{\text{b}^{\text{*}}\text{×}\text{ L}^{\text{*}}}$$

Total soluble solids (TSS) were measured with a refractometer PR101-a (Atago, Japan) according to the manufacturers’ instructions and two measurements were conducted per fruit.

Total fruit weight and peel weight were measured by an electronic balance (Sartorius, Germany), and the edible rate was calculated using the equator below.

$$\text{Edible rate (\%) =}\frac{\text{Total fruit weight (g)}-\text{Peel weight (g)}}{\text{Total fruit weight (g)}}\text{×100}$$

Transverse diameter and longitudinal diameter were measured using a digital caliper (SATA, China), and the shape index was calculated by the equator below.

$$\text{Shape index =}\frac{\text{Longitudinal diameter (mm)}}{\text{Transverse diameter (mm)}}$$

The granulation index was evaluated based on Chen et al. (1995) and Nie et al. (2020). Granulation level was evaluated according to the portion of the granulated area and was determined as: No granulation, level 0; granulated area ≤ 10%, level 1; 10% ≤ granulated area ≤ 25%, level 2; 25% ≤ granulated area ≤ 50%, level 3; granulated area ≥ 50%, level 4. The granulation index was calculated according to the following formula.

$$\text{Granulation index =}\frac{\text{∑Granulation level × Number of fruit in this level}}{\text{The highest granulation level (level 4) × Total fruit number}}$$

The juice sacs from five groups were collected, weighed by an electronic balance (Sartorius, Germany) and recorded as *W_0_*. Then the fresh samples of juice sacs were frozen in liquid nitrogen and lyophilized using a vacuum freeze dryer (Virtis, USA). The lyophilized juice sacs were weighed again and then recorded as *W_1_*. Three replicates were performed for each group, with 15 fruit per replicate. The water content and dry matter content were calculated using the equators below.

$$\text{Water content (\%) =}\frac{\text{W}\text{0}-\text{W}\text{1}}{\text{W}\text{0}}\text{×100}$$

$$\text{Dry matter content (\%) =}\frac{\text{W}\text{1}}{\text{W}\text{0}}\text{×100}$$

1.2 Determination of Soluble Sugars, Organic Acids, Amino Acids by GC-MS

The gas chromatography-mass spectrometer (GC-MS) method was applied for the determination of soluble sugars, organic acids and amino acids. Sample preparation and derivatization were performed based on the method of Kang et al. (2022). Then the GC-MS analysis was performed on Agilent 7980B GC and Agilent 7000C MSD (Agilent Technology, USA). One microliter of each sample was injected into a HP-5ms column (30 m × 0.25 mm i.d., 0.25 μm; Agilent Technology, USA), with a split ratio of 10:1. The temperature of the injector was 250 °C, with helium as carrier gas at a flow rate of 1 mL/min. The total run time was 55 min. The ion source temperature was set as 230 °C, with an ionization voltage of -70 eV. Mass spectra were recorded with a mass-to-charge ratio range of 30-550 m/z and a scanning speed of 0.4 scans/s. The contents of soluble sugars, organic acids and amino acids were calculated by the standard curves of authorized standards, which were purchased in Beiing Solarbio Science & Technology Co., Ltd.

1.3 Cell Wall Components Qualification

For the determination of cell wall components contents, the juice sacs from each group were lyophilized and ground into fine powder. Three replicates were conducted for each group, with 15 fruit per replicate.

Alcohol insoluble solids (AIS) were extracted according to the method of Dietz and Rouse (1953). In brief, the lyophilized flesh of 1 g was homogenized in 25 mL of 95% ethanol, and then incubated three times in a water bath at 90 °C, 30 min each time. Then the samples were centrifuged at 3000 × g for 5 min to remove supernatant. The final residue was dried overnight at 60 °C and weighed. The AIS content was calculated by the following formula.

$$\text{AIS content }\left（ \text{\%} \right）\text{=}\frac{\text{Dried residue weight (g)}}{\text{Lyophilized flesh weight (g)}}\text{ ×100}$$

The sequential extraction of water-soluble pectin and protopectin from AIS was performed by the method of Dong et al. (2008). Approximately 200 mg of AIS powder from each group was extracted twice with 25 mL distilled water , with a 50 °C water bath for 30 min each time. After centrifugation at 3000 × g for 5 min, the supernatant was collected as water-soluble pectin extracts. The residue was extracted with 25 mL of H_2_SO_4_ (0.5 mol/L) and incubated in a water bath at 85 °C for 10 min. Then the solution was centrifugated at 3000 × g for 5 min to obtain protopectin extracts. For the measurement of pectins, 0.6 mL of H_2_SO_4_ (containing 0.48% sodium tetraborate) was added to 0.1 mL of water-soluble pectin and protopectin extracts, respectively. After hydrolyzation for 12 h, 10 µL of 0.15% M-hydroxy biphenyl was added to the solution. The absorbance of solutions was detected at 530 nm by a microplate reader (BIO-RAD, USA). The contents of pectins were calculated based on a standard curve of galacturonic acid and expressed as mg/g dry weight (DW).

The extraction and measurement of cellulose and hemicellulose were based on the method of Zhou et al. (2011) with slight modifications. Briefly, the residue from the protopectin extraction was collected and homogenized in 10 mL of 100 mmol/L NaBH_4_ (resolved by 4 mmol/L NaOH). After ultrasonic-assisted extraction for 6 h, the supernatant was obtained by centrifugation at 3000 × g for 5 min to get the hemicellulose. The remaining residue was washed with distilled water twice in order to obtain the cellulose. For the hydrolyzation of hemicellulose and cellulose, 12 mL of 2mol/L H_2_SO_4_ and 3 mL of 80% H_2_SO_4_ were added separately, followed by a boiling water bath for 5 h. Finally, 3 mL of 0.25% anthrone-H_2_SO_4_ reagent was added to the cellulose and hemicellulose extracts, respectively. After incubating in a 90 °C water bath for 15 min, the absorbance of solutions was detected at 620 nm. The contents of cellulose and hemicellulose were calculated by a standard curve of glucose, which were expressed as mg/g DW.

The measurement of lignin content was carried out according to Chang et al. (2008). In brief, 15 mg of AIS powder was extracted with 1.5 mL of 90% dimethyl sulfoxide and oscillated overnight to remove the starch. After centrifugation at 10000 × g for 5 min, the remaining residue was washed six times with 1 mL of 70 % ethanol , followed by 1.5 mL of acetone. Then the residue was dried at 65 °C overnight to obtain de-starched AIS. After that, 1 mL of 25% acetyl bromide was added to de-starched AIS and then incubated in a water bath at 70 °C for 1 h, followed by adding 5 mL of acetic acid. Then 300 µL of the supernatant was collected, followed by adding 400 µL of NaOH (1.5 mol/L) and 300 µL of hydroxylamine hydrochloride (0.5 mol/L), respectively. The absorbance of solutions at 280 nm was recorded. The lignin content was calculated by acetyl bromide soluble lignin (ABSL) assay and expressed as ABSL%.

1.4 Cell Wall Components Labeling in the Histologic Section of Juice Sac

For the observation of transparency and lignin distribution of juice sacs, juice sacs from five groups were cut transversely and longitudinally. Then the juice sacs were observed and imaged using stereomicroscope Discovery. V8 (Zeiss, Germany). For the lignin staining, 1% phloroglucinol (dissolved in 95% ethanol) was added to the juice sacs for 3 min, followed by adding 30% HCl. After staining, the juice sacs were photographed again and the images were exported by ZEN (Version 2.6).

Paraffin sections were used for the staining of pectin, cellulose and lignin. The preparation of paraffin sections was performed according to the protocol of Li et al. (2017) with slight modifications. Briefly, juice sacs from five groups were collected and fixed in formaldehyde solution (containing 45% ethanol) for 48h. After dehydration by ethanol and transparency by xylene, the juice sacs were filled with 65 °C wax for 2 h and then embedded in a carton for wax block making. The slice thickness for pectin and cellulose staining was 6 µm, and for lignin staining was 8 µm, respectively. After being stored at 62 °C overnight, the sections were dewaxed by xylene and ethanol, respectively. The detailed procedures of paraffin sections can be found at Supplementary Materials.

The immunofluorescence staining of pectin was based on the method of Chudzik et al. (2018). Briefly, the EDTA (pH 9.0) (ZSGB-BIO, Beijing) were used for antigen repairing through boiled water bath. After being washed by PBS (pH 7.4), the sections were blocked in 1% bovine serum albumin (Solarbio, Bejing) for 1 h at room temperature. JIM 5 and JIM 7 (PlantProbes, UK) were adopted as primary monoclonal antibodies for the labelling of low-esterified and high-esterified pectin, respectively. The sections were incubated in primary antibodies at 4 °C overnight, with a dilution of 1: 20 by PBS (pH 7.4). Then the sections were washed with PBS (pH 7.4) for 3 times, with 5 min one time. Alexa Flour 488-goat anti-mouse IgG(H+L) (Thermo Fisher Scientific, USA) was used as secondary antibody. After diluted 1: 200 by PBS (pH 7.4), the secondary antibody was added to the sections and incubated at 20 °C for 1 h. The sections were washed with PBS (pH 7.4) for 3 times. Finally, the sections were sealed with Antifade Mounting Medium (Solarbio, Beijing) and observed under EGFP channel, with the excitation wavelength of 488 nm.

The cellulose was stained with Calcofluor White (Coolaber, Beijing) according to the manufactures’ instructions. After repaired by EDTA (pH 9.0), the sections were washed with PBS (pH 7.4) for 3 times. Then the Calcofluor White (diluted 1:1 with distilled water) and 10% KOH were added to the sections and then incubated in darkness for 5 min, followed by washing with PBS (pH 7.4) for 3 times. Finally, the sections were sealed with Antifade Mounting Medium and observed under DAPI channel, with the excitation wavelength of 405 nm.

For lignin staining, sections were stained with 0.1% toluidine blue solution for 5 min, and then washed with distilled water for 5 min. After staining, the sections were sealed with neutral gum and observed under brightfield. The images of paraffin sections were all captured using VS200 Research Slide Scanner (Olympus, Japan) and exported by OlyVIA (Version 3.3).

1.5 Phytohormones Metabolites Profiling

The extraction and determination of phytohormones were carried out by the method of Chen et al. (2013) with slight modifications. Briefly, approximately 1 g of peel or pulp was frozen in liquid nitrogen and ground into fine powder. Then 50 mg of the sample was extracted with 0.5 mL of methanol/water/formic acid (15:4:1, V/V/V) at 4 °C overnight. 10 μL of internal standard mixed solution (100 ng/mL) was added for the quantification. The mixture was vortexed for 10 minutes, followed by centrifugation at 12,000 × g under 4 °C for 5 min. After that, the extracts were evaporated to dryness by nitrogen gas steam, reconstituted in 100 µL of 80% methanol (V/V), and then filtered through a 0.22 μm filter (Anpel, China) for further analysis.

The detection of phytohormones was performed by Wuhan Metware Biotechnology Co., Ltd (Wuhan, China) based on the liquid chromatography-electrospray ionization-tandem mass spectrometry (LC-ESI-MS/MS) system (LC-ESI-MS/MS system (HPLC, ExionLC™ AD; MS, Applied Biosystems 6500 Triple Quadrupole). Three replicates were conducted for each group, with each replicate from at least 15 fruit. The contents of phytohormones were calculated using the external standard method and expressed as ng/g on a fresh weight basis.
